# Spindle Cell Squamous Cell Carcinoma of the Scalp Treated With Pembrolizumab Cisplatin and 5-Fluorouracil

**DOI:** 10.7759/cureus.20242

**Published:** 2021-12-07

**Authors:** Ali M Alzahrani, Bander Al Mutari, Anas Alzahrani, Abdelhafeeth Alkhodaidi, Gaaem Yahya

**Affiliations:** 1 Oncology, King Fahad Medical City, Riyadh, SAU; 2 College of Medicine, Imam Muhammad Ibn Saud Islamic University, Riyadh, SAU

**Keywords:** cutaneous carcinosarcoma, pembrolizumab, scalp, spindle cell carcinoma, immunotherapy

## Abstract

Cutaneous spindle cell squamous cell carcinoma (SpSCC) of the head and neck is a very rare tumor. It is an aggressive variant of squamous cell carcinoma. The usual treatment of the localized disease is surgery with or without radiotherapy. No standard treatment for metastatic disease although some case reports had reported the effectiveness of programmed cell death protein 1 (PD-1) blockade as a possible treatment.

We are reporting a 57-year-old Arabic female presented with metastatic scalp spindle cell squamous carcinoma, who was treated with three lines of chemotherapy. She received pembrolizumab, cisplatin, and 5-fluorouracil for three cycles but did not respond, the pembrolizumab was dropped and we added cetuximab for three more cycles but did not respond also. She had a partial response to doxorubicin single agent as a third line. Our case showed resistance to pembrolizumab and cetuximab combined with chemotherapy regimens which are both considered as standard treatments for the classical squamous cell carcinoma of the head and neck, but there was a partial response to single-agent doxorubicin.

## Introduction

Spindle cell squamous cell carcinoma (SpSCC) is a very rare type of cancer, representing less than 3% of all head and neck epithelial cancer [1]. It has aggressive metastatic or locally advanced behavior [2]. It is considered by many authorities as a subtype of squamous cell carcinoma while others state that it should be considered as a different identity [3]. Primary cutaneous spindle cell squamous cell carcinoma was first identified by Yang et al. [4]. It usually occurs in sun-exposed areas, in previously exposed areas to ionizing radiation, or in immunocompromised individuals. No standard systemic treatment is available for metastatic disease. The anti-programmed cell death protein 1 (anti-PD-1) antibody cemiplimab was approved on September 28, 2018, by the Food and Drug Administration (FDA) for the treatment of metastatic or locally advanced cutaneous SCC, following practice-changing trials [5,6].

The FDA had also approved the anti-PD-1 human monoclonal antibody pembrolizumab with or without platinum-based chemotherapy as a standard first-line treatment for metastatic and locally advanced/recurrent squamous cell carcinoma (SCC) of head and neck origin [7]. Information regarding the applicability of these regimens to the rare spindle cell variant for the head and neck squamous cell carcinoma is unfortunately limited. We are presenting the response and tolerability of such protocol in this rare entity and our recommendation for such treatment in future trials with larger numbers if possible.

## Case presentation

**Figure 1:**
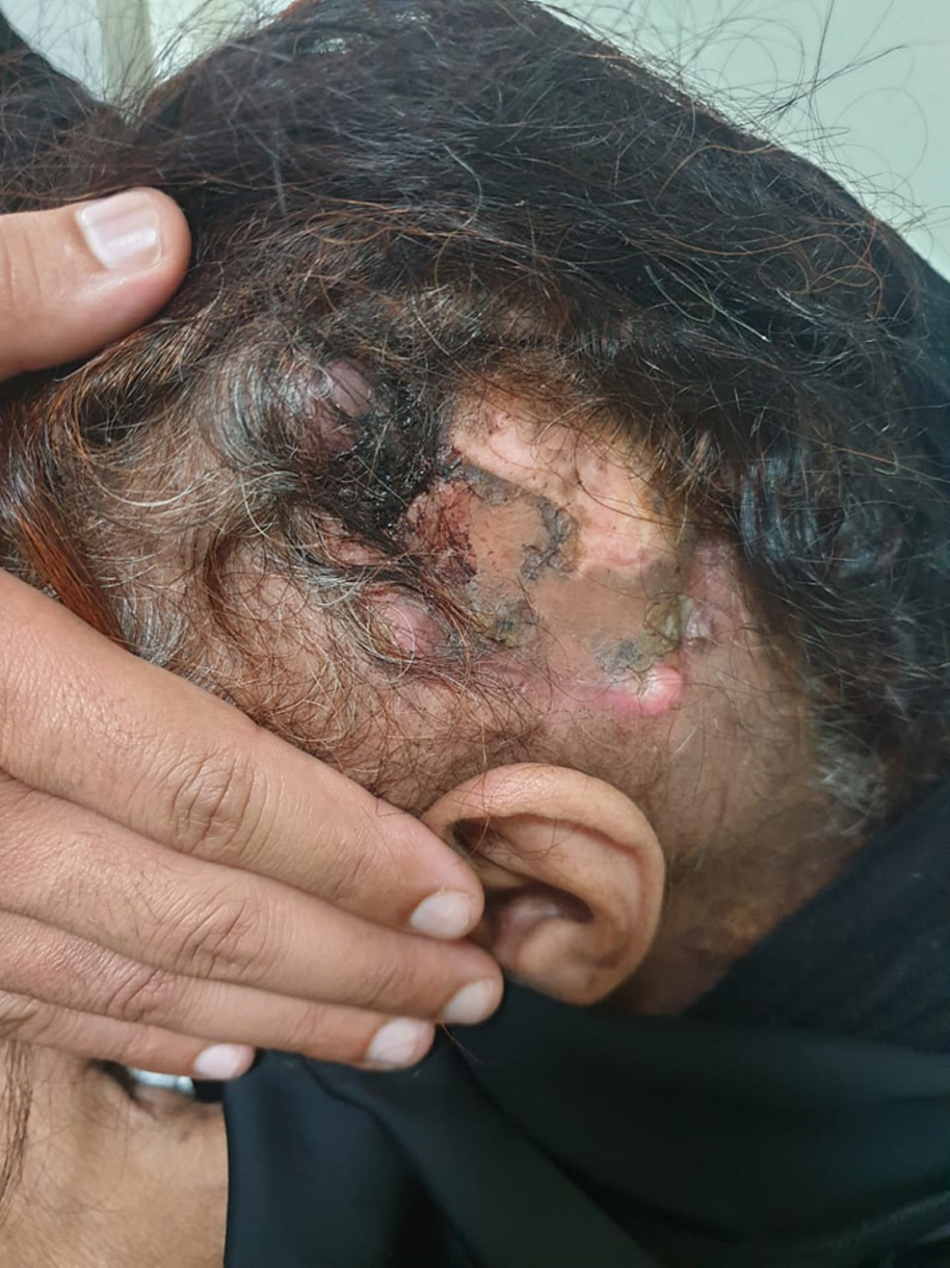
Nodular lesions at the margins of the previous surgical scar.

We are reporting a 57-year-old Arabic female who noticed a lesion of the right side of the scalp in July 2019, it was not tender and measured approximately 1.5 cm. She underwent a partial excision at a local hospital with a preliminary diagnosis of a benign lesion. The final pathology was a malignant lesion. No official pathology report was available for review. She refused further management. The lesion progressed again rapidly within one month; she presented herself to another private hospital where more radical surgery was carried out with left selective neck dissection, this was done on October 16, 2019. The pathology report was spindle cell squamous cell carcinoma, all margins were negative and all the lymph nodes were negative. She was referred to King Fahad Medical City, Riyadh, Saudi Arabia, in November 2019 for further management. She was a fit lady, not a smoker or drinker, however, was a known diabetic on oral hypoglycemic drugs for 10 years with fair glycemic control. She was complaining of mild pain at the site of the lesion with no other associated symptoms. On examination, there were two small nodules at the margins of the previous surgical scar. The superior nodule measure 1 cm x 1.5 cm and the other was 1.5 cm x 2 cm at the inferior surgical margin (Figure 1).

**Figure 2:**
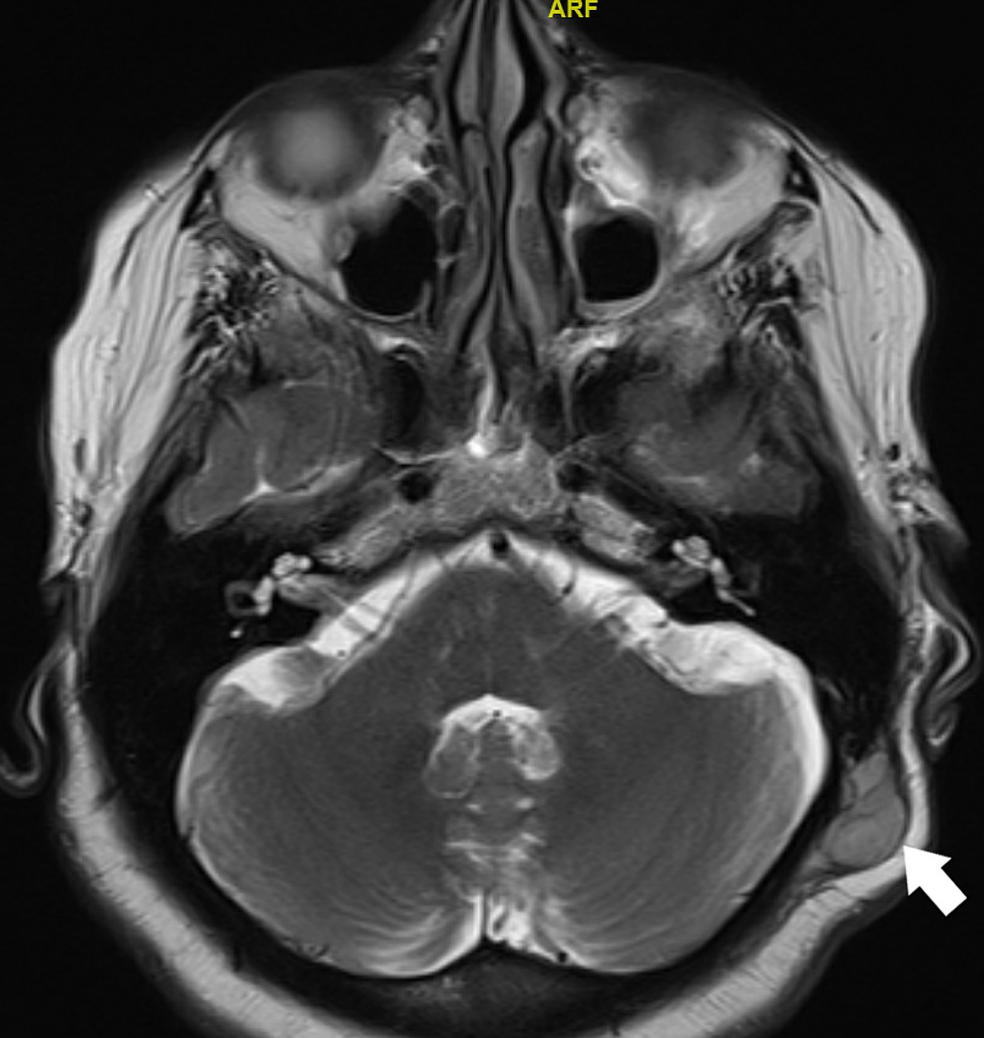
MRI showing the lower lesion (white arrow).

**Figure 3:**
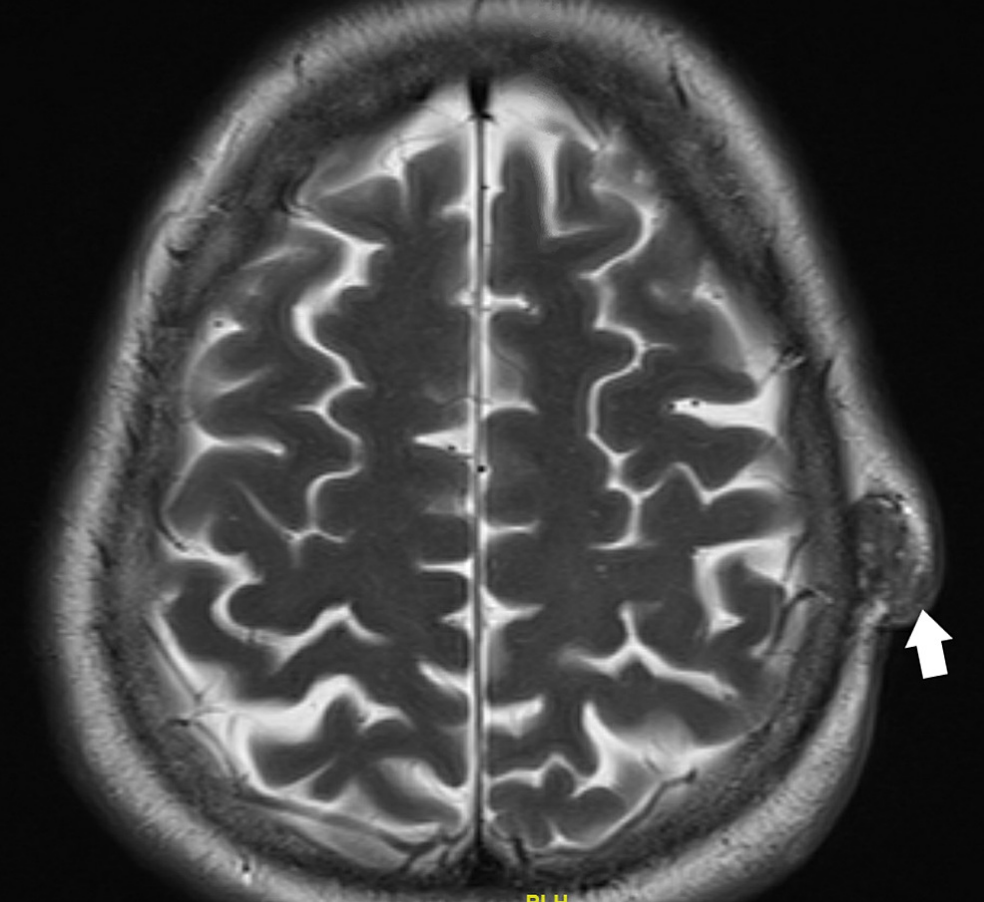
MRI showing the upper lesion (white arrow).

**Figure 4:**
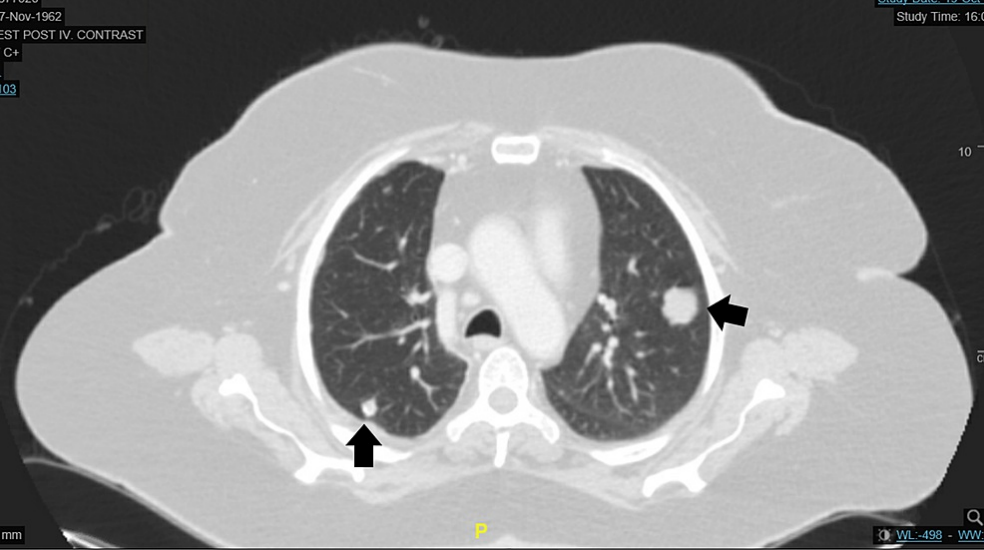
CT scan of chest showing multiple lung metastases (black arrows).

She underwent full reevaluation with magnetic resonance imaging (MRI) of the head and neck and computer tomography (CT) of the chest, abdomen, and pelvis. The MRI showed that there was evidence of residual/recurrent soft tissue tumor at the superior and inferior aspects of the surgical bed. The first lesion at the high left parietal area measured 1.2 cm x 2 cm x 2.1 cm, it demonstrated features of internal hemorrhage. The other lesion noted at the left occipital region measured about 1.2 cm x 2.3 cm x 2.5 cm. Both lesions demonstrate post-contrast enhancement. There was no evidence of underlying bony involvement and no intracranial extension was seen. A few small lymph nodes were noted at the posterior neck on the left side (Figures 2, 3). The CT chest showed multiple lung metastases (Figure 4).

**Figure 5:**
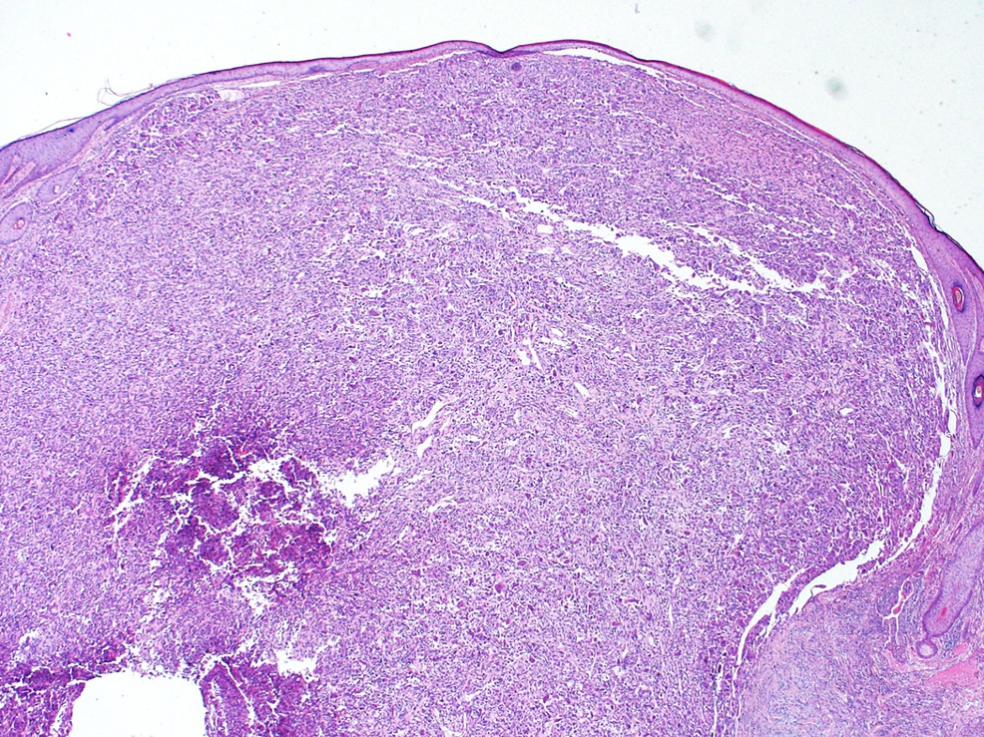
The image is showing spindle cell carcinoma, small islands and, cords of squamous cell carcinoma and dense proliferation of neoplastic spindle cells.

The histopathology review has shown malignant spindle cell neoplasm, poorly differentiated/sarcomatoid squamous cell carcinoma with perineural invasion but without lymphovascular invasion (Figure 5). Immunohistochemistry staining showed positive staining of vimentin, desmin, and cluster of differentiation (CD) 10. The cytokeratin staining, cytokeratin (CK)-Pan, CK5/6, P63, and CK7 were focally positive. S100, human melanoma black (HMB)-45, CD117, paired-box gene 8 (PAX-8), estrogen receptors (ER), progesterone receptors (PR), CK20, gross cystic disease fluid protein 15 (GCFP-15), CD34, CD1a, myoblast determination protein 1 (MyoD1), and myogenin were all negative. All of the above were supporting the diagnosis.

She was discussed in the tumor board and the decision was to give palliative radiotherapy to the local lesion and to start palliative systemic chemotherapy. She was fit with Eastern Cooperative Oncology Group (ECOG) performance status of one. After a full discussion of the possible benefits and side effects, it was decided to start her on pembrolizumab, cisplatin, and 5-fluorouracil protocol since the main histopathological component was squamous cell carcinoma. Programmed death-ligand 1 (PD-L1) testing was sent abroad since the local laboratories were still not accredited at that time. She received three cycles with good tolerance but the reevaluation CT scan showed progression of the lung metastases. At that time the PD-L1 testing came back as negative. We substituted pembrolizumab with cetuximab. Further three cycles were given but after the third cycle, she started to have shortness of breath. Chest x-ray and CT scan showed further progression with right pleural effusion. She was admitted for pleural drainage and pleurodeses. After stabilization, she was given single-agent doxorubicin. After three cycles of doxorubicin, she had symptomatic improvement with the partial radiological response, but she refused further treatment because she felt more fatigued during chemotherapy. She was kept under follow-up with palliative, symptomatic care at her regional hospital. She progressed further and died after three more months.

## Discussion

Most of the SpSCC patients who present with localized head and neck lesions are usually treated with surgery [8]. In the early stages, surgery alone has an excellent outcome. In later stages, the usual treatment is the combination of surgery and radiotherapy although there are some reports indicating radioresistance [9]. Although there are some studies that challenged the concept of radioresistance of spindle cell carcinoma and reported similar responses and outcomes to the classical squamous cell carcinoma [10,11]. Another study had reported the possible detrimental effects of radiotherapy when added to surgery in terms of survival [12].

Two retrospective series showed poor survival outcomes, especially those with oral cavity, sinonasal and oropharyngeal sites, compared to SCCs at similar sites [13,14]. The median overall survival (OS) of 8.9 months was found in a study [14]. For the early-stage group (stages I and II), the three years survival rate of 100% was reported, while those with stages III and IV had one-year survival rate of 9% and three years survival rate was 0%, respectively [14].

Using the surveillance, epidemiology, and end results (SEER) database, one study had compared 4382 cases of classical sinonasal SCCs with 328 cases of other pathological subclassifications. Five-year disease-specific survival (DSS) was as follows: 84.7% for verrucous cell carcinoma, 61.87% for papillary cell carcinoma, 56.2% for basaloid cell carcinoma, 45% for classical squamous cell, 32% for SpSCC, and 15% for adenosquamous carcinoma. A total of 65.6% of patients with SpSCC were treated with a combination of surgery and radiotherapy compared to 40.4% with conventional SCC [10].

Two reported cases with SpSCC of the tongue with locally advanced disease (T4N2M0 and T4aN1M0) were managed with a combined modality treatment, i.e., radical surgery followed by adjuvant chemoradiotherapy. The first case had developed lung metastasis five months after surgery while the second case was disease-free two months after surgery [15].

Although the epidermal growth factor receptor (EGFR) is expressed by >90% of conventional SCC and possibly in 70% of SpSCC, EGFR-specific therapies may not be ideal for SpSCC patients according to some reports [16]. Our case had received anti-EGFR cetuximab with no response.

One case report had suggested that anti-PD-1 therapy with pembrolizumab may be an effective and well-tolerated treatment for patients with SpSCC with metastasis to the CNS [17]. But our case did not show any obvious response when we used pembrolizumab with cisplatin and 5-fluorouracil. Another case report had suggested that 5-fluorouracil is an effective treatment in spindle cell carcinoma of the nasal cavity but our case did not show the same [18].

Recently, pembrolizumab plus platinum and 5-fluorouracil were approved as first-line treatment for recurrent or metastatic head and neck squamous cell carcinoma (HNSCC) and pembrolizumab monotherapy for PD-L1-positive recurrent or metastatic HNSCC in a phase 3 trial [7]. We used the same protocol for SpSCC but unfortunately, our patient did not respond. The possible reasons are either because of the negative PD-L1 testing or because of the inherent resistance of this variant to immunotherapy. Our case was showing partial response to doxorubicin single agent as a third line which is well-known chemotherapy for sarcoma, possibly indicating the response of the malignant spindle cells part of the tumor.

## Conclusions

Our patient with metastatic spindle cell carcinoma of the scalp has shown resistance to pembrolizumab, cisplatin, 5-fluorouracil and cetuximab, cisplatin, 5-fluorouracil regimens which are standard treatments for the classical squamous cell carcinoma of the head and neck. There was a partial response to doxorubicin which is usually effective in sarcoma cases.
